# Susceptibility of acute myeloid leukemia cells to ferroptosis and evasion strategies

**DOI:** 10.3389/fmolb.2023.1275774

**Published:** 2023-09-25

**Authors:** Hanyun Zhang, Chunjie Sun, Qi Sun, Ye Li, Chao Zhou, Changgang Sun

**Affiliations:** ^1^ College of First Clinical Medicine, Shandong University of Traditional Chinese Medicine, Jinan, China; ^2^ College of Traditional Chinese Medicine, Shandong University of Traditional Chinese Medicine, Jinan, China; ^3^ State Key Laboratory of Quality Research in Chinese Medicine and Faculty of Chinese Medicine, Macau University of Science and Technology, Macau, China; ^4^ Department of Oncology, Weifang Traditional Chinese Hospital, Weifang, China; ^5^ College of Traditional Chinese Medicine, Weifang Medical University, Weifang, China

**Keywords:** ferroptosis, acute myeloid leukemia, iron metabolism, ROS, lipid metabolism

## Abstract

Acute myeloid leukemia (AML) is a highly aggressive hematologic malignancy with a 5-year survival rate of less than 30%. Continuous updating of diagnostic and therapeutic strategies has not been effective in improving the clinical benefit of AML. AML cells are prone to iron metabolism imbalance due to their unique pathological characteristics, and ferroptosis is a novel cell death mode that is dominated by three cellular biological processes: iron metabolism, oxidative stress and lipid metabolism. An in-depth exploration of the unique ferroptosis mechanism in AML can provide new insights for the diagnosis and treatment of this disease. This study summarizes recent studies on ferroptosis in AML cells and suggests that the metabolic characteristics, gene mutation patterns, and dependence on mitochondria of AML cells greatly increase their susceptibility to ferroptosis. In addition, this study suggests that AML cells can establish a variety of strategies to evade ferroptosis to maintain their survival during the process of occurrence and development, and summarizes the related drugs targeting ferroptosis pathway in AML treatment, which provides development directions for the subsequent mechanism research and clinical treatment of AML.

## 1 Introduction

AML is a cancer originating from the hematopoietic stem cell myeloid lineage. It is characterized by the rapid proliferation of primitive cells and their replacement of hematopoietic tissue within the bone marrow, ultimately causing disruption to the normal bone marrow microenvironment. Within this pathological context, hematopoietic progenitor cells undergo clonal expansion, resulting in a blockage of their differentiation and maturation at various stages of development. The incidence of AML has increased annually over the past few decades, and the mortality rate has remained high owing to its aggressive nature and high recurrence rate. Currently, hematopoietic stem cell transplantation (HSCT) and the traditional “3 + 7”procedure, which was developed in the 1970s, are still used to treat AML. Demethylation medicines, IDH inhibitors, FLT3 inhibitors, and BCL-2 inhibitors are just a few medications that have recently been developed as a result of an improved understanding of the pathogenic pathways causing AML, including genetic characteristics and epigenetic changes. The introduction of these medications has effectively enhanced patient survival and quality of life by bringing AML treatment into the realm of precision therapy (Dohner et al., 2021). However, there has been no breakthrough in the overall survival rate of patients with AML. Therefore, new treatment strategies for AML are urgently needed.

In 2012, Stockwell introduced the concept of “ferroptosis” and described it as a type of cell death caused by uncontrolled lipid peroxidation and secondary membrane damage (Gao et al., 2016). Mechanistically, reactive oxygen species (ROS), phospholipid peroxides (PLOOH), and other metabolites that are inevitably produced during cell growth and differentiation react with polyunsaturated fatty acids (PUFAs), which are susceptible to peroxidation, causing membrane peroxidation, loss of permeability, and an increase in toxic products, which further destroys the cellular structure (Jiang et al., 2021). There is great potential in the treatment of cancer and other diseases that are closely related to ferroptosis by inducing ferroptosis in cells (Stockwell, 2022). Currently, the induction of ferroptosis to cause cancer cell death in hematological tumors, including AML, is considered a promising new therapeutic strategy (Zhang et al., 2022a). When AML is first diagnosed, it is commonly accompanied by iron overload, manifested mainly by elevated ferritin levels due to insufficient erythropoiesis and inflammatory signaling, which is aggravated by red blood cell infusion in late chemotherapy, providing AML cells an environmental basis for ferroptosis to occur (Weber et al., 2020). High expression of SLC7A11 and GPX4 are risk factors for AML patients and may also serve as prognostic markers (Han et al., 2022). In addition, Erastin, RSL3 were found to increase the anticancer potency of anthracyclines and cytarabine and inhibit AML cell proliferation (Skouta et al., 2014; Yu et al., 2023). Altered AML cell metabolism, genetic mutations, and dependence on mitochondria make AML cells appear to be highly susceptible to ferroptosis. However, the ensuing evasion strategy established by AML cells adds to the uncertainty regarding the occurrence of ferroptosis.

This paper provides a review of the core mechanism of ferroptosis and its impact on AML. It also highlights why AML is susceptible to ferroptosis and discusses strategies to prevent its occurrence. In addition, the paper discusses the use of iron-death-related drugs in AML treatment. The future application prospects of ferroptosis in AML treatment are also explored, along with potential challenges and corresponding suggestions.

## 2 Ferroptosis core mechanisms

**FIGURE 1:**
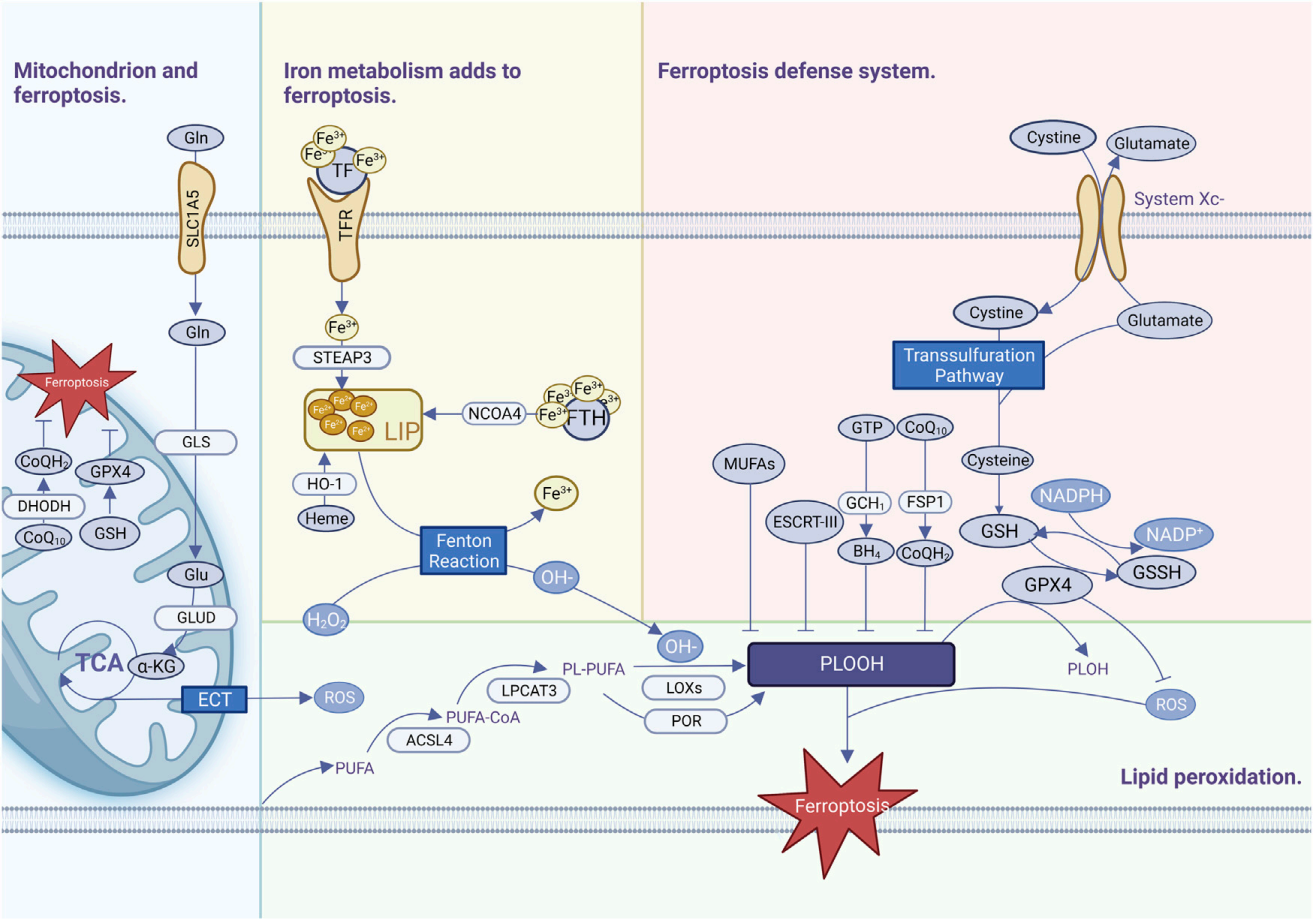
Ferroptosis occurrence and defense mechanisms.

Ferroptosis is commonly thought to be one of the oldest and most prevalent types of cell death and has been observed not only in mammals but also in plants, protozoa, and fungi (Hadian and Stockwell, 2020). Ferroptosis has been defined as a highly complicated process requiring the coordinated engagement of various metabolisms and pathways, with three primary features of iron metabolism imbalance, lipid peroxide formation, and antioxidant system breakdown (Figure 1).

### 2.1 Abnormal iron metabolism

Iron is an essential trace element involved in many physiological activities in the human body, is extremely oxidizing, generates excess ROS through the Fenton reaction, and is an important cofactor involved in the synthesis of enzymes responsible for lipid peroxidation and oxygen homeostasis (Holbein and Lehmann, 2023). Iron is usually bound to a complex such as heme (a prosthesis of numerous proteins such as hemoglobin), iron-sulfur clusters (a cofactor for many enzymes), or ferritin (a specialized iron storage protein) (Stockwell, 2022). A small amount of intracellular iron that is not bound as a complex is called the labile iron pool (LIP), which is highly reactive and oxidizable. Fe^3+^ in food is reduced to Fe^2+^ by duodenal cytochrome B, which is transferred to LIP via the divalent metal transporter (DMT1) (Donovan et al., 2000). Ferritin is recognized by nuclear receptor coactivator 4 (NCOA4), which directs ferritin into the autophagosome for degradation to release Fe^2+^ into the LIP (Yu et al., 2022). The LIP generates a large number of free radicals via the Fenton reaction, which damages cellular lipids and proteins due to the instability and easy redox potential of hydroxyl radicals (Dixon et al., 2012). The proteins and pathways involved in iron uptake, storage, transport, and transformation are diverse, and affect the susceptibility of cells to ferroptosis to varying extents (Stockwell, 2022). Despite a growing understanding of iron metabolism and ferroptosis, it is still impossible to quantify the amount of iron required by tumors and normal tissues, and it is difficult to measure the level of unstable iron pools.

### 2.2 Lipid peroxidation reaction

Uncontrolled lipid peroxidation is a typical characteristic of ferroptosis. The lipid peroxidation substrates of ferroptosis are PUFAs, which are important components of cell membrane organelles and have unstable carbon–carbon double bonds with a strong affinity for free radicals. The hydrogen atoms between the double bonds are easily oxidized by free radicals and subsequently prone to cascade-type reactions, which expand the scope of ferroptosis (Yang et al., 2016). PUFAs break down and oxidize. Subsequently, they form lipid radicals, lipid hydroperoxides, reactive aldehydes, and other toxic substances that cause further damage to cells (Xin and Schick, 2023). Acyl-CoA synthetase long-chain family member 4 (ACSL4) promotes the activation of PUFAs such as arachidonoyl (AA) and adrenal to synthesize PUFA-CoA (Kagan et al., 2017), and then lysophosphatidylcholine acyltransferase 3 (LPCAT3) binds PUFA-COA to membrane phospholipids (PLs) to catalyze the formation of phospholipids containing PUFA-PLs (Kagan et al., 2017). Eventually, PUFA-PLs are oxidized by iron and lipoxygenases (LOXs) to generate PUFA-PL-OOH, which trigger ferroptosis (Kagan et al., 2017). ROS produced by electron leakage from the mitochondrial electron transport chain (ETC.) may also contribute to lipid peroxidation during ferroptosis in some situations (Gao et al., 2019). Monounsaturated fatty acyl (MUFA) can compete with PUFAs to bind PL via ACSL3 to prevent erastin-induced ferroptosis, or exogenous MUFA can reorganize intracellular lipid metabolism so that excess lipids are stored in organelles such as triglycerides (TAG), a form of lipid that is less susceptible to oxidative reactions (Liang et al., 2022).

### 2.3 Antioxidant defense mechanisms

To meet high proliferative demands, cancer cells need to integrate multiple metabolic pathways including amino acid synthesis, the tricarboxylic acid cycle, oxidative phosphorylation, and iron metabolism. These active and complex metabolic pathways are usually accompanied by an imbalance of oxidative stress, which makes a robust ferroptosis defense mechanism essential for cancer cells. The XC-GPX4-GSH pathway plays a central role in ferroptosis defense, and system XC- is an amino acid transporter consisting of two key components: disulfide-linked solute carrier family 3 member 2 (SLC3A2) and solute carrier family 7 member 11 (SLC7A11) (Koppula et al., 2021). System XC exchanges extracellular cystine and intracellular glutamate in a 1:1 ratio. After entry into the cell, cystine is converted to cysteine, which is then converted to glycine with glutamate by cysteine-glutamate ligase, and then reduced to glutathione (GSH) catalyzed by glutathione synthetase (GSS) (Jiang et al., 2021). GPX4-GSH scavenges free radicals in the body and maintains intracellular free radical stability. Glutathione is the most important antioxidant in cells and a cofactor of GPX4. GPX4 is a selenoprotein that contains glutathione and selenium to maintain its activity (Liu et al., 2023a). GPX4 is the most important intracellular anti-lipid peroxidase, which uses GSH to participate in the LOOH reduction reaction to catalyze the conversion of harmful lipid peroxides to harmless lipid alcohols and prevents the occurrence of ferroptosis. GPX4 activity can be suppressed by inhibiting the XC upstream system (Zhang et al., 2021). Erastin, a ferroptosis inhibitor, acts on the XC-inhibiting cystine uptake system, leading to inhibition of intracellular GSH synthesis (Dixon et al., 2012). RSL3, a ferroptosis inhibitor, acts on GPX4 to inactivate it, resulting in a large accumulation of intracellular lipid peroxides, ultimately leading to the onset of ferroptosis (Zheng et al., 2023). In summary, the XC-GPX4-GSH system is a complex network in which various molecules affect ferroptosis indirectly or directly via the XC-GPX4-GSH system. In addition to ferroptosis, it affects apoptosis, pyroptosis, autophagy, and other cell-death mechanisms (Kang et al., 2023).

Some cancers resist ferroptosis after GPX4 inactivation, suggesting the existence of additional defense mechanisms. Recently, FSP1-coenzyme Q10 (COQ10) (Bersuker et al., 2019), DHODH-COQ10 (Mao et al., 2021), GCH1-tetrahydrobiopterin (BH4) (Vasquez-Vivar et al., 2022), and endosomal sorting complexes in retrograde transport-III (ESCRT-III) have been identified as ferroptosis defense mechanisms that are not dependent on the XC-GPX4-GSH system and function in parallel with the XC-GPX4-GSH system for parallel defense against ferroptosis (Pedrera et al., 2021).

### 2.4 Mitochondria and ferroptosis

The mitochondrial OXPHOS system is at the center of cellular metabolism and is critical for energy production in eukaryotic cells (Vercellino and Sazanov, 2022). Ferroptosis is promoted by metabolites during mitochondrial biosynthesis, e.g., ferroptosis is driven by the generation of ROS, ATP, and/or PUFA-PL produced during the TCA cycle. Inhibition of the mitochondrial TCA cycle or the, ETC., attenuates mitochondrial membrane potential hyperpolarization and inhibits lipid peroxide accumulation and ferroptosis (Gao et al., 2019). Accordingly, the ferroptosis defense system in mitochondria is mainly composed of GPX4-GSH, dihydroorganic acid dehydrogenase (DHODH)-COQ10 (Zheng and Conrad, 2020), while mitochondrial ferritin overexpression can also regulate iron homeostasis in mitochondria to inhibit Erastin-induced ferroptosis (Wang et al., 2016).

## 3 Susceptibility of AML cells to ferroptosis

### 3.1 Metabolic characteristics of AML cells confer susceptibility to ferroptosis

**FIGURE 2:**
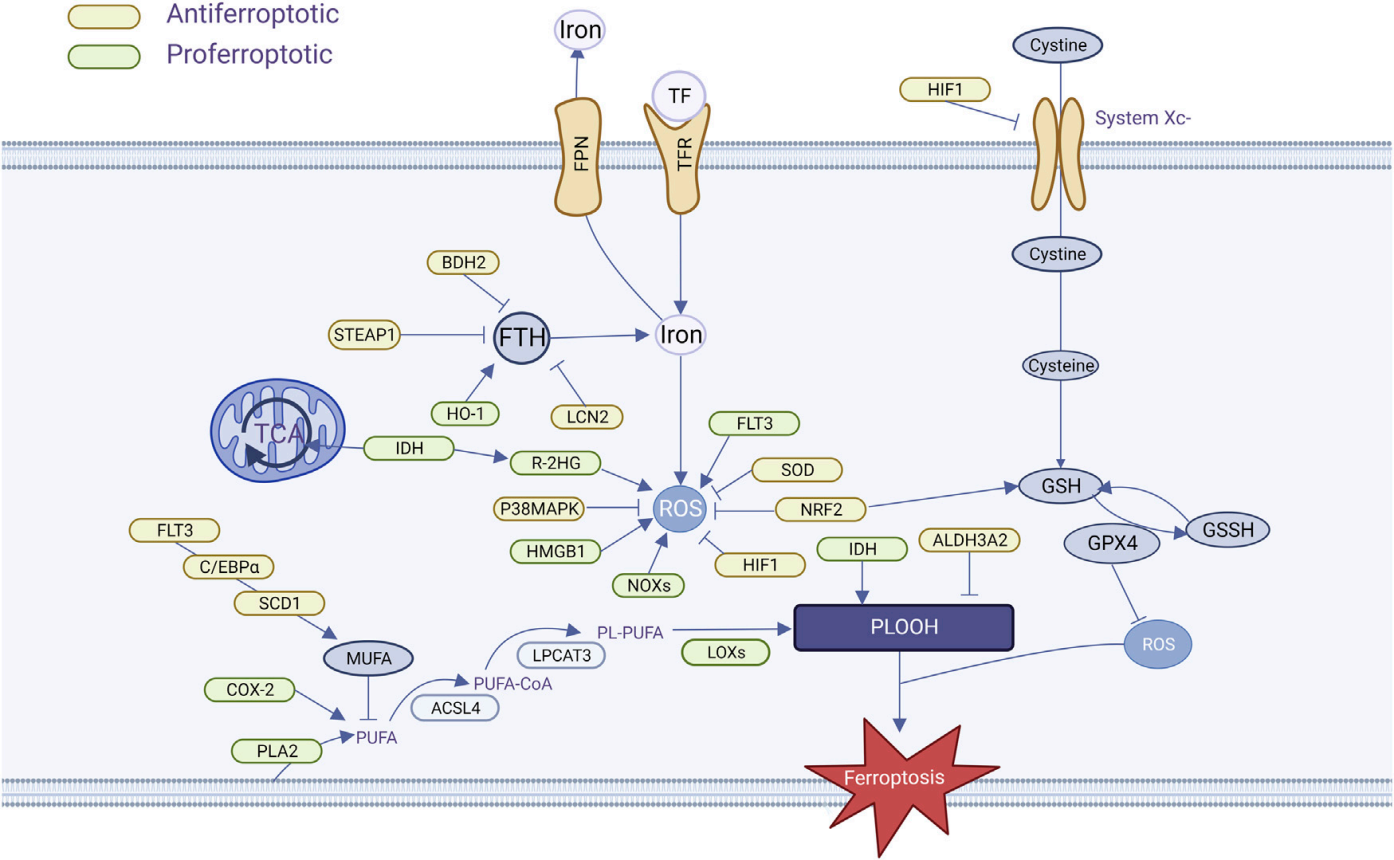
AML cells regulate ferroptosis pathways.

AML is distinguished by the presence of an abnormally high number of immature myeloid cells in the bone marrow that are unable to differentiate and mature properly, proliferate rapidly, or occupy space for normal cell survival. To maintain their highly proliferative and highly differentiated state, AML cells have growth characteristics of extremely high energy demand, susceptibility to oxidative stress, and accumulation of metabolites, which provide the conditions for ferroptosis to occur (Figure 2).

#### 3.1.1 Iron overload and iron regulation imbalance

AML cells are particularly susceptible to iron overload. Patients with AML generally have high serum ferritin levels at initial diagnosis, with levels positively correlated with tumor load, which drop to normal levels after remission, and elevated levels may present with recurrence (Lebon et al., 2015). The most common cause of iron overload is multiple infusions of red blood cells during chemotherapy. One unit of red blood cells contains approximately 200 mg of iron (Wang et al., 2021), part of the iron in the transfused erythrocytes is recycled and stored in the tissues by macrophages in the liver and spleen, and the heme iron that cannot be stored is released outside the cell, increasing the amount of unstable iron pool. Measurement of iron overload by means of biomagnetism, magnetic resonance imaging, or organ biopsy has revealed the presence of iron overload in patients with MDS/AML and has led to a better understanding of the characteristics of their iron metabolism (Jensen, 2004). Heme iron overload damages bone marrow hematopoietic stem cells and affects bone marrow hematopoietic function via the ROS pathway (Lu et al., 2013; Chai et al., 2015). In turn, the decreased hematopoietic function becomes dependent on blood transfusions, creating a vicious cycle. In addition, increased heme iron increases the risk of infection-related complications, and Petzer et al. observed a strong correlation between Aspergillus growth and heme in AML patients undergoing allogeneic HSCT (Petzer et al., 2019). Usually, in order to eliminate the negative effects of iron overload, iron stingers such as Deferasirox and Deferiprone are applied clinically to reduce the iron load in AML patients. Chang et al. found that Deferasirox has a pronounced antileukemic effect, which induces apoptosis in AML cells by stinging unstable iron pools, generating large amounts of ROS, and blocking cell signaling pathways (Chang et al., 2017). We hypothesized that iron stingers such as Deferasirox may hinder the onset of AML cell ferroptosis when applied, which has been demonstrated in hepatocellular carcinoma (Jomen et al., 2022).

Transferrin (TF) and transferrin receptor (TFR) bind to form a complex that is involved in intracellular iron transport and is the main pathway for iron uptake by cancer cells, which was found to be essential for the induction of cellular ferroptosis by glutamine depletion assays (Gao et al., 2015). There is much evidence that AML cells are highly bound to TF and are highly expressed in AML cells (Yeh et al., 1984), but the specific effect on AML cells is unknown. TFR play important roles in ferroptosis. By upregulating TFR, cells with RAS mutations have increased intracellular iron content, making them more sensitive to elastin-induced ferroptosis, while knocking out TFR makes them less sensitive to erastin-induced ferroptosis (Yang and Stockwell, 2008). Two TFRs have been identified: TFR1 and TFR2. Iron enters the cell mainly via TFR1 and is transported via NCOA4 receptor-mediated endocytosis. TFR1 has been validated as a marker of ferroptosis (Feng et al., 2020). TFR1 expression is elevated in AML cells compared to normal cells, and TFR cell expression is elevated as malignant cell differentiation decreases (Liu et al., 2014; Lyons and Pappas, 2019). In addition, it has been suggested that TFR1 is associated with anemia, thrombocytopenia, and complex cytogenetics in AML (Wu et al., 2016), but not with poor AML prognosis. In bone marrow specimens from AML and MDS patients, however, the researchers found that high TFR2 mRNA expression had a relatively favorable prognosis, suggesting that TRF2 may alter iron metabolism and increase AML cell sensitivity to chemotherapy (Nakamaki et al., 2004; Di Savino et al., 2017).

The ferritin-ferromodulin (FPN) system is an important regulator of iron homeostasis, and is associated with redox control. Ferritin, which consists of FTH and FTL, inhibits iron efflux, keeps the LPI pool stable, and inhibits ferroptosis. Ferritin deficiency decreased the regulatory effects of SLC7A11 and causes ferroptosis (Fang et al., 2020). Compared with normal HSCs, FTH and FTL were overexpressed in both AML cells and LSCs, and FTH overexpression was usually accompanied by NF-κB-related gene expression, which reduced chemosensitivity (Bertoli et al., 2019). Thus, elevated ferritin levels are indicative of disease progression. FPN is the sole protein responsible for controlling intracellular iron efflux. When FPN is overexpressed, it reduces he amount of iron within cells and hinders the growth of cancer cells. Therefore, FPN could potentially serve as a vulnerability for cancer cells that heavily rely on iron. Studies have shown that FPN expression is reduced in AML compared to normal cells, up to 100,000-fold in some cell lines. The researchers concluded that low-FPN cells retain more iron and have an increased susceptibility to ROS. Follow-up experiments verified this hypothesis via *ex vivo* experiments that the application of iron oxide nanoparticles to AML cells with low FPN showed better anti-leukemic activity, which could explain the greater sensitivity of AML cells with low FPN to chemotherapeutic drugs. In addition, iron-regulator expression in AML is associated with an inflammatory milieu, and AML patients with low levels of FPN may have reduced FPN expression due to greater autocrine or paracrine secretion of inflammatory cytokines (such as IL-6) (Vela et al., 2018). Increased intracellular iron levels, coupled with an imbalance in iron metabolism, further increase the vulnerability of AML cells to ferroptosis.

#### 3.1.2 Increased reactive oxygen species

As an inhomogeneous group of molecules and free radicals, reactive oxygen species (ROS) are frequently involved in various cellular processes. It is frequently found to be elevated in a variety of cancer types (Trachootham et al., 2009), and AML is no exception (Zhang et al., 2014; Hole et al., 2011). In AML, ROS are generally acquired via altered metabolic pathways such as xanthine oxidoreductase, non-coupled nitric oxide (NO) synthase (NOS), cytochrome P450 monooxygenase (CYP), cyclooxygenase (COX), and NADPH oxidase (NOX). The high frequency of ROS overproduction in cancer and leukemia strongly suggests that ROS are associated with diseases pathogenesis of these diseases is inseparable (Hole et al., 2011). In hematological malignancies, ROS plays a dual role in disease progression (Udensi and Tchounwou, 2014). In the presence of high levels of ROS, chemotherapeutic agents inhibit tumor growth and cause apoptosis by producing high levels of ROS (Khoshtabiat et al., 2016). In contrast, low ROS levels protected AML cells from apoptosis and promoted cell proliferation, survival, growth, migration, and drug resistance (Kaweme et al., 2020).

Nicotinamide adenine dinucleotide phosphate (NADPH) oxidase (NOXS), a class of membrane-bound enzyme complexes that catalyze the production of superoxide, contributes to iron-dependent lipid peroxidation accumulation during ferroptosis (Yang et al., 2020), and in 60% of the patients, AML cells constitutively produce a large number of extracellular superoxides through the NOx family of NADPH oxidases, such as NOX1, NOX2, NOX4, and NOX5, with NOX2 being the main source of superoxide in AML (Irwin et al., 2013), with evidence showing strong expression of NOX4 in a subpopulation of patients with high ROS (Hole et al., 2013). Accordingly, reactive oxygen species produced by the NOx family hhavebeen linked to leukemic progression, DNA damage, and cellular transformation in many ways (Jayavelu et al., 2016a; Jayavelu et al., 2016b).

High mobility group box 1 (HMGB1) is a transcriptional protein sensitive to oxidative stress and is involved in multiple processes of cancer development, such as cell proliferation, angiogenesis, cell migration, and adhesion (Chen et al., 2023). Some researchers have found that HMGB1 is a central factor necessary for the occurrence of ferroptosis. Reducing ROS levels by SOD1 depletion or NAC significantly affected HMGB1 transport, whereas inhibition of HMGB1 expression prevented erythroid-induced GPX4 degradation, TFR1 expression, and subsequent ferroptosis. This suggests that HMGB1-dependent ROS is directly involved in the occurrence and maintenance of erythroid-induced ferroptosis in AML cells (Ye et al., 2019).

#### 3.1.3 Altered lipid metabolism

AML cells exhibited enhanced cholesterol utilization and synthesis rates, decreased total cholesterol and cholesterol-to-phospholipid ratios, increased phosphatidylcholine and phosphatidylinositol, and increased unsaturated fatty acids (Vitols et al., 1990). Similar to these results, Pabst et al. found depletion of total plasma fatty acids and cholesterol in AML cells, but an increase in certain free fatty acids such as sphingolipids, choline phosphate, and a decrease in triglycerides and cholesteryl esters may be caused by enhanced fatty acid oxidation in AML cells (Pabst et al., 2017). Importantly, free fatty acids (such as arachidonic acid 20:4 n-6 and the corresponding precursors gamma-linolenic acid 18:3 n-6 and 8,11,14-eicosatrienoic acid 20:3 n-6) were found to be increased in AML cells compared to normal samples, and successively, transcript levels of soluble phospholipase A2 (PLA2) isoforms IB and X were found to be both in AML cells showed upregulation (Fiancette et al., 2009). This finding is crucial because the enzymatic activity of PLA2 catalyzes the release of AA from membrane phospholipids, which in turn generates lipoxygenase and its derived lipid mediators. LOXs drive ROS to oxidize AA and AdA-PE, and the subsequent generation of AA- and AdA-PE-OOH causes ferroptosis (Kagan et al., 2017). AA is important for the induction of ferroptosis, and AML cells with high AA expression are more malignant.

Cyclooxygenase 2 (COX-2) is a key enzyme in prostaglandin biosynthesis with dual roles as a peroxidase and dioxygenase that can promote PUFA lipid peroxidation in organelles involved in ferroptosis. The inhibition of COX-2 is an effective way to alleviate ferroptosis caused by ferritin overload. Multiple experiments have demonstrated that COX-2 production can be induced with LPS in AML cellsand stimulated HL-60 cells also express COX-2 (Vincent et al., 2008a; Puhlmann et al., 2005), which may promote ferroptosis.

### 3.2 Genetic mutations in AML cells confer susceptibility to ferroptosis

Somatic mutations in isocitrate dehydrogenases 1/2 (IDH1/2, respectively) are found in approximately 20% of patients with AML. Both mutants conferred novel morphological activity for the NADPH-dependent reduction of ketoglutarate (KG) to the oncogenic metabolite (R)-2-hydroxyglutarate (2HG) (Dang et al., 2009). Interestingly, one study found that IDH1 and IDH2 mutant AML cells produce the metabolite R-2HG that rapidly increases intracellular ROS, which in turn affects NF-κB protein stability and transcriptional activity (Chen et al., 2016). A study found that IDH-mutant AML cells exhibit enhanced mitochondrial oxidative metabolism and that fatty acid oxidation in IDH-mutant cells is further increased with increased TCA cycle products (Stuani et al., 2021). A recent experiment identified a new metabolic vulnerability in AML cells mutated in IDH1, which significantly disrupts lipid metabolism and is characterized by a lack of monoacylglycerides and lysophospholipids and an increase in acylcarnitine species. This lipid metabolism abnormality is more pronounced in IDH1 than in IDH2 (Thomas et al., 2023).

Fms-like tyrosine kinase 3 (FLT3) is mutated in approximately 90% of AML patients, and FLT3-ITD is a common mutation in FLT3, accounting for approximately 20% of AML patients, and is more malignant. Sabatier et al. suggested that FLT3-ITD mutant AML cells regulate fatty acid synthesis by modulating downstream CCAAT/en-hancer-binding protein α (C/EBPα)a and steroyl-CoA desaturase (SCD, mentioned below). When C/EBPα was inhibited in FLT3-ITD mutant AML cells, SCD expression was downregulated, resulting in decreased MUFA formation and increased production of PUFAs, which led to iron cell death (Sabatier et al., 2023). FLT3-ITD mutated cell lines with upregulated heme oxygenase-1 (HO-1) and nuclear factor erythroid 2-related factor 2 (Nrf2) expression produce higher levels of ROS (Kannan et al., 2022), DNA oxidation, and double-strand breaks compared to wild-type FLT3 patients (Sallmyr et al., 2008; Godfrey et al., 2012). The major source of ROS in FLT3-ITD mutant AML cells is the NOx family. Excessive ROS accumulation has signaling functions that promotes cell proliferation and migration, thus contributing to leukemic cell transformation (Godfrey et al., 2012; R et al., 2011). Targeting the Nrf2-HO-1 antioxidant pathway using the ferroptosis-related drug brusatol improved the inhibition of FLT3-ITD mutant cell proliferation (Kannan et al., 2022). IDH1/2 and FLT3-IDT mutations in AML lead to abnormalities in ROS, lipid metabolism, and increased susceptibility to ferroptosis, but it was not found whether these two mutations affect iron metabolism, which can be further explored as a direction for future research.

### 3.3 Mitochondria-dependent conferral of ferroptosis susceptibility in AML cells

Independent of the mutational spectrum and cytogenetic influences, mitochondrial metabolism dominates AML metabolism, is the main source of cellular ROS, and is the main site of OXPHOS (Gao et al., 2021). Many studies have shown that mitochondrial energy metabolism is significantly altered in AML cells, influencing the occurrence of ferroptosis in AML cells in terms of bioenergetics, biosynthesis, and ROS regulation.

While reducing OXPHOS could provide a survival advantage for cancer cells, AML cells do not want to do so. Compared to hematopoietic stem cells, AML cells show an increased dependence on mitochondrial metabolism and OXPHOS, and increased mitochondrial mass (Skrtic et al., 2011). Additionally, AML cells lack metabolic flexibility and struggle to meet their energy demands by inhibiting fatty acid oxidation to promote glycolysis. Consequently, AML cells depend heavily on mitochondrial OXPHOS for energy supply (Gao et al., 2021). This facilitates the onset of ferroptosis: connecting cancer cell metabolism to OXPHOS makes highly aggressive cancer cells more sensitive to ferroptosis by disrupting glycolysis (Zdralevic et al., 2018). However, OXPHOS overactivity also renders AML cells more vulnerable to resistance against cytarabine, suggesting the presence of a more robust antioxidant system that protects AML cells (Farge et al., 2017). In addition, overactivation of the mitochondrial protease CLPP can degrade respiratory chain proteins mainly conforming to the I subunit, leading to an increase in ROS in mitochondria (Ishizawa et al., 2019). Meanwhile, during the rapid growth phase of cancer cells, more iron is translocated into mitochondria to meet the exuberant energy demand of cancer cells (Duan et al., 2022). Due to the unique metabolic dependence on mitochondria, AML cells are more prone to ferroptosis.

## 4 AML cells escape ferroptosis

**FIGURE 3:**
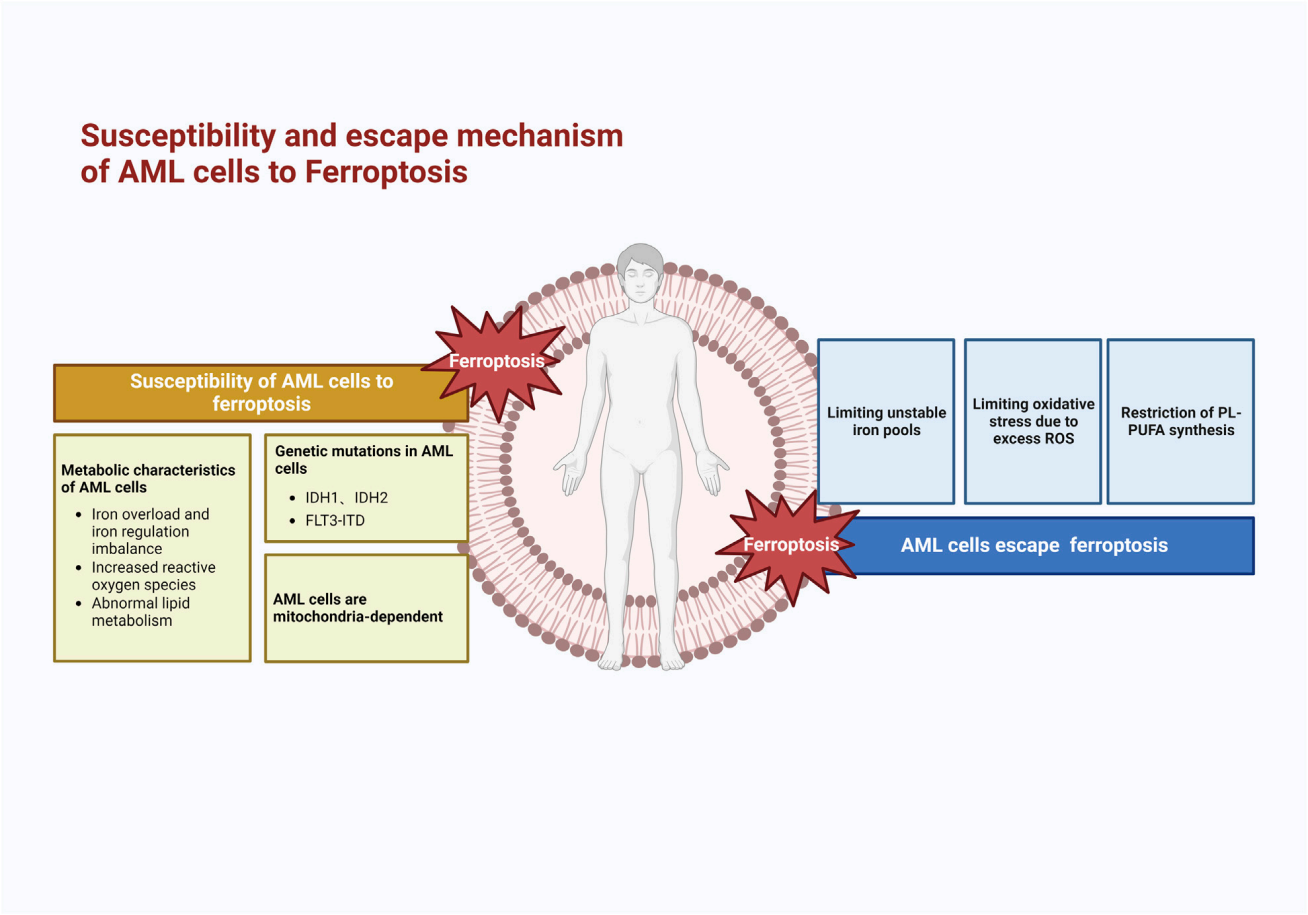
Mechanism of susceptibility to ferroptosis and escape mechanism of AML cells. AML cells are highly susceptible to ferroptosis because of their metabolic and genetic alterations; therefore, AML cells develop evasion strategies such as limiting unstable iron pools, oxidative stress, and lipid peroxidation reactions.

The susceptibility of AML cells to ferroptosis is a barrier to their survival and development. To avoid the risk of ferroptosis when lipid peroxidation occurs to a certain extent, AML cells construct a number of iron avoidance strategies to cope with ferroptosis, including (1) limiting the unstable iron pool, (2) limiting oxidative stress due to excessive ROS, and (3) limiting PUFA synthesis (Figure 3), which makes it difficult to induce ferroptosis in AML cells. Therefore, it is necessary to understand the ferroptosis evasion strategies in AML cells.

### 4.1 Limiting LIP

Elevated serum ferritin, an iron storage protein, is commonly found in most patients with new-onset AML, and this abnormality is not dependent on transfusion. It has been observed that increased ferritin levels lead to resistance to cytarabine, thus increasing the risk of HSCT recurrence and decreasing overall survival (Bertoli et al., 2019). Furthermore, patients with high ferritin expression have shown lower response rates when treated with decitabine for new-onset AML (Park et al., 2017). It is hypothesized that ferritin might contribute to methylation resistance. The presence of ferritin limits the expansion of LIP, reduces the formation of ROS, and prevents ferroptosis from occurring to a certain extent. How to break the iron homeostasis and convert the large amount of stored iron in ferritin into unstable free iron making it more likely to induce ferroptosis is a direction that can be studied in the future.

Lipid transport protein-2 (LCN2) belongs to a family of lipid transport proteins that are widely involved in the development of many types of inflammatory malignant diseases (Chi et al., 2020). This mechanism has been shown to function as an iron regulatory protein that is strongly bound to trivalent iron (Kjeldsen et al., 2000). Patients with many hematologic tumors, including AML, have expression of LCN2 (Bouchet and Bauvois, 2014; Candido et al., 2014), which is required for the BCR-ABL-induced mouse leukemia model and is involved in damaging normal hematopoietic cells and inducing tissue invasion by leukemia cells (Leng et al., 2008). Multiple experiments have found lower levels of LCN2 expression in bone marrow specimens from AML patients than in normal subjects (Candido et al., 2014; Yang et al., 2013a). Surprisingly, LCN2 expression levels gradually increased with remission in AML patients, and refractory AML patients showed low LCN2 levels (Yang et al., 2013a). High LCN2 expression in bone marrow is associated with a favorable prognosis, especially in patients with FLT3 mutations (Yang et al., 2013b). BDH2 is a novel cytosolic-type 2-hydroxybutyrate de hydrogenase involved in lipid metabolism pathways and iron transport, which also participates in the mitochondrial and tricarboxylic acid cycles (Guo et al., 2006). Devireddy et al. found that BDH2 accompanies 2,3-DHBA production during enterobactin synthesis, and contributes to iron transport and apoptosis via the LCN2-mediated pathway (Devireddy et al., 2010). In contrast to LCN2, BDH2 high expression is an independent poor prognostic factor in AML patients, with a pro-proliferative effect on AML cells and reduced sensitivity to chemotherapeutic agents (Yang et al., 2013a). It can be further hypothesized that cells with high LCN2 expression increase iron content through its iron transport function, leading to lipid peroxidation and thus ferroptosis, and high BDH2 expression prevents cytoplasmic iron overload and reduces the likelihood of ferroptosis.

STEAP3, an iron reductase within the STEAP family, mediates the conversion of trivalent to divalent iron and is an important regulator of ferroptosis. Although STEAP1 has no iron-reducing function (Ohgami et al., 2005), STEAP1 expression in Ewing tumors correlates favorably with cellular reactive oxygen species, which stimulates the production of oxidative stress-sensitive and invasive genes, which is consistent with earlier research: Thyroid epithelial cell proliferation is aided by increased STEAP1 expression (Pan et al., 2008). Another study found that STEAP1 covalently localizes to TFRC to participate in iron metabolism (Ohgami et al., 2006), suggesting that it also plays an important role in ferroptosis. In AML, researchers found that STEAP1 overexpression is associated with poor prognosis, and presumably, STEAP1 favors proliferation and survival in AML (Chen et al., 2021).

### 4.2 Limiting oxidative stress due to excess ROS

Collapse of the redox system is one of the triggers for the occurrence of ferroptosis. To limit reactive oxygen species, AML cells establish an antioxidant system to prevent ferroptosis from occurring.

NRF2 is a redox-dependent transcription factor involved in the pathogenesis and drug resistance mechanisms of many tumors, and affects AML cells to evade ferroptosis in two main ways. First, NRF2 overexpression can activate antioxidant function to help AML cells resist chemical damage. NRF2 is significantly expressed in high tumor load and drug-resistant AML, and is highly expressed in patients with poor prognosis (Hu et al., 2022). Correspondingly, overexpression of NRF2 also induced gene instability-dependent drug resistance in AML (Liu et al., 2021). By reducing ROS, NRF2 renders AML resistant to cytarabine and zorubicin (Karathedath et al., 2017). Further studies revealed that inactivation of the NRF2 pathway leads to reduced production of antioxidants (e.g., SOD2), which shifts the ratio of GSH/GSSG to the oxidized form, thereby increasing AML cellular chemosensitivity (Niu et al., 2021). Second, NRF2 regulates transcriptionally almost all genes related to ferroptosis, including regulation of GPX4 activity and glutathione production, which affects NADPH regeneration, glutathione-dependent thioredoxin and peroxiredoxin reduction (Anandhan et al., 2020). NRF2 also regulates genes related to iron metabolism, preventing the accumulation of free iron to avoid ferroptosis (Harada et al., 2011). Liu et al. identified a novel mechanism by which in AML Liu et al. found a new mechanism that in AML, GPX4 is an important target gene of NRF2, and activation of NRF2 can cause GPX4 to be overexpressed in AML cells, thus avoiding ferroptosis (Liu X. et al., 2023). It has been shown that the use of ferroptosis inducers generates large amounts of ROS in AML cells, which resist oxidative stress by activating NRFR/HO-1 through nuclear translocation of the NRF2 protein, and that downregulation of NRF2 expression markedly increases the killing effect of ferroptosis inhibitors (Ali et al., 2016). Therefore, NRF2 is a very important pathway target for AML cells to evade ferroptosis.

HO-1, an enzyme that catalyzes the conversion of heme to bilirubin, is a downstream target of Nrf2 (Kerins and Ooi, 2018). HO-1 is also involved in accelerating erastin-induced ferroptosis (Chang et al., 2018). The Nrf2-HO-1 pathway also has a regulatory effect on intracellular iron concentration. Nrf2 degrades HO-1 to biliverdin/bilirubin, nitric oxide, and divalent iron, and HO-1-induced ferroptosis is achieved by degradation of ferritin, which increases free iron (Tomiotto-Pellissier et al., 2018). However, HO-1 in ferroptosis appears to be a double-edged sword, depending on the metabolic state of the tumor and the tumor microenvironment. HO-1 appears to play a role in evading ferroptosis and protecting AML cells. Wei et al. demonstrated that HO-1 levels are elevated in AML patients (Wei S. et al., 2015). Numerous experiments have shown that HL60-resistant cells have higher expression of HO-1 than HL-60-sensitive cells, identifying a correlation between HO-1 overexpression and AML resistance, and subsequent experiments have hypothesized that it may be through a ROS-dependent pathway thereby allowing AML cells to escape chemotherapy-induced apoptosis (Zhe et al., 2015; Heasman et al., 2011). After the inhibition of HO-1 expression, resistant and refractory cell lines showed sensitivity to cytarabine and erythromycin. This is consistent with the results of another team’s experiments: downregulation of HO-1 by gene silencing and the classical HO-1 inhibitor ZnPPIX, which found that HO-1 silencing greatly sensitized AML cells to Zoerythromycin (Wei et al., 2014; Wei S. X. et al., 2015).

Large amounts of ROS burst in new-onset AML cells, and excess ROS or the resulting manifestation of antioxidant defense deficiency leads to cellular damage and enhanced antioxidant signaling; thus, normal cells activate stress protein kinases (SAPKs) including P38-MAPK mitogen-activated protein kinase (MAPK), which responds to reactive oxygen stress through phosphorylation and thus affects the cell cycle (Cuadrado and Nebreda, 2010; Bulavin and Fornace, 2004). It has been shown that AML cells will adapt to a high ROS state and downregulation of P38-MAPK does not affect normal cellular activity. Because excessive ROS block normal hematopoiesis (Juntilla et al., 2010; Ito et al., 2004), AML cells also gain an advantage in competition with normal cells through the ROS paracrine mode (Hole et al., 2013).

Superoxide dismutases (SODs) can catalyze the conversion of superoxide to oxygen and hydrogen peroxide, control the levels of reactive oxygen and reactive nitrogen in the body, limit the potential toxicity of these substances while controlling reactive substance signaling, and are the first line of defense against oxygen radicals (Wang et al., 2018). SODs are commonly expressed in other hematological tumors and are highly expressed in AML (Chen and Kan, 2015). In addition, other factors significantly interfere with SODs expression, such as a slight increase in SOD1 levels and a significant decrease in SOD2 in AML cells with aberrant expression of the transcription factor GATA-1 (Lu et al., 2007). This adds to the complexity of the problem, but what remains constant is that the detoxifying superoxide action of SODs is essential in AML (Huang et al., 2000), and inhibition of SODs levels can lead to cell death (Chen and Kan, 2015).

Hypoxia is a hallmark of the tumor microenvironment, and the hypoxia-inducible factor (HIF) transcript is a major regulator of hypoxia (Fuhrmann and Brune, 2022), which controls tumor glycolysis, angiogenesis, and cell proliferation and migration by affecting downstream targets (Chen et al., 2022). Hematopoietic stem cells in the bone marrow require a hypoxic environment to maintain quiescence and preserve their self-renewal potential. HIF-1 increases the transcription of SLC7A11 and HO-1 to prevent ferroptosis (Feng et al., 2021; Yuan et al., 2022), unlike HIF-2, which promotes lipid peroxidation to induce ferroptosis (Singhal et al., 2021). In AML cells, both HIF1 and HIF-2 are more highly expressed than in normal cells (Wang et al., 2011; Rouault-Pierre et al., 2013), and their silencing induces apoptosis in LSCs and disrupts AML cells repair reconstruction in xenograft mice (Wang et al., 2011). A study on MDS/AML found that HIF-1 reduced ROS in iron-overloaded MDS/AML cells, prevented ferroptosis, and prompted cells to enter the S-phase-and G2/M-phase cell cycles. In contrast, the inhibition of HIF-1 expression in an iron overload model resulted in a significant increase in ROS, causing cellular injury (Zheng et al., 2017). Thus, HIF-1 is important for regulating ROS in AML cells and circumventing the excessive accumulation of oxides.

### 4.3 Restriction of PL-PUFA synthesis

LOXs are non-heme iron dioxygenases (Kuhn et al., 2005) named ALOX, and are widely believed to be central players in the prolipid peroxidation process, of which ALOX5, ALOX12, and ALOX15 have been shown to trigger ferroptosis (Shah et al., 2018). In AML cells, the expression of ALOX5/12/15 transcripts is lower than that in normal freshly isolated plasma (Vincent et al., 2008b). This may be a means for AML cells to avoid ferroptosis, limiting PUFA synthesis and peroxidation. However, the mechanism that inhibits reduced ALOX production is not known.

Stearoyl-CoA desaturase 1 (SCD1), an enzyme that catalyzes the rate-limiting step of monounsaturated fatty acid synthesis in cancer cells, is an important regulator of lipid metabolism. Normally, SCD1 overexpression in cancer cells promotes monounsaturated MUFAs production and significantly reduces lipid peroxide levels (Yi et al., 2020). SCD is a ferroptosis-protective gene in AML, and CIRCZBTB46 protects AML cells from ferroptosis by upregulating SCD1 expression in a mouse xenograft model. CIRCZBTB46 is an important circRNA that is frequently expressed in AML cells and regulates cell proliferation and ferroptosis (Long et al., 2023). Recently researchers have found that the FLT3-C/EBPα-SCD axis affects fatty acid synthesis oxidation in AML patients with FLT3-ITD mutations, and that inhibition of FLT3 mutations leads to decreased expression of SCD1, resulting in an increase in PUFAs compared to MUFAs, and ultimately sensitization of AML cells to ferroptosis (Sabatier et al., 2023). Thus SCD1 plays an important role in AML cells in avoiding the occurrence of ferroptosis by limiting PUFA synthesis.

Aldehyde dehydrogenase 3a2 (ALDH3A2), an enzyme responsible for detoxifying fatty aldehydes and producing C16-18 fatty acids, is expressed in leukemic stem cells with specific metabolic vulnerabilities. Aldehydes are metabolites of oxidative phosphorylation and nucleotide synthesis in cancer that are produced by lipid peroxidation and form the basis of ferroptosis (Monteiro et al., 2022). Yusuf et al. found a unique role for ALDH3A2 in reducing the susceptibility of leukemic stem cells to ferroptosis. The researchers inhibited GPX4 activity and increased the number of ROS, following which increased ALDH3A2 depletion and ferroptosis occurred. They found that ALDH3A2 and GPX4 act in parallel: in AML, simultaneous inhibition of ALDH3A2 and GPX4 activity has a far greater cell-killing effect than inhibition of GPX4 activity alone. ALDH3A2 and GPX4 differ in that ALDH3A2 is involved in fatty acid synthesis and, via its enzymatic reactions, both replenish damaged fatty acids and limit continued fatty acid damage. Thus, compared to GPX4, ALDH3A2 inhibited ferroptosis in AML cells more strongly.

## 5 Ferroptosis-related drugs in AML

In recent years, drugs for the treatment of AML ferroptosis, including small molecule inhibitors, natural compounds, and nano-delivery, are being developed and utilized, and new advances are being made, and here we summarize the mechanisms and targets of drugs targeting AML ferroptosis (Table 1).

**TABLE 1:** Ferroptosis inducers for AML treatment.

Ferroptosis inducer	Target	Mechanism	Study
Erastin	SLC7A11	Inhibition of NFE2L2-SLC7A11 pathway	Yu et al. (2015)
RSL3	GPX4	Inhibition of GPX4 activity	Yang et al. (2016)
APR-246	GSH	Inhibition of GSH synthesis	Birsen et al. (2022)
ATPR	ROS	Activation of ROS-autophagy-lysosomal pathway	Du et al. (2020)
Neratinib	Mechanism unknown	Activation of autophagy‐dependent ferroptosis	Ma et al. (2022)
Sulfasalazine	GSH	Inhibition of SLC7A11	Zheng et al. (2022)
Iron complexes	Apo-TF	Induction of oxidative stress	(Baecker et al., 2020; Sagasser et al., 2019)
Artemisinin derivatives	AMPK/mTOR/p70S6k	Activation of AMPK/mTOR/p70S6k signaling pathway	Liu et al. (2023c)
Typhaneoside	AMPK	Activation of AMPK signaling	Zhu et al. (2019)

### 5.1 Ferroptosis inducer

Ferroptosis inducers can be divided into two categories: those targeting systemic XC, such as Erastin and those targeting GPX4, such as RSL3. Erastin directly inhibits systemic XC-inhibited cystine uptake and reduces GSH synthesis. It has a good ferroptosis-inducing effect in many cancers (Hu et al., 2023; Xu et al., 2023). In AML cells, erastin inhibits cell growth and proliferation and has good antitumor potential (Yu et al., 2015). The ferroptosis inducer RSL3 directly inhibits GPX4 activity by covalently binding to selenocysteine (Yang et al., 2016). Studies have confirmed that the application of RSL3, an inhibitor targeting GPX4, in AML, triggers multiple cell deaths in AML cells, including ferroptosis. RSL3 can also be combined with chemotherapeutic agents such as cytarabine and adriamycin to improve the response rate to chemotherapy (Yu et al., 2015).

### 5.2 APR-246

A previous study found that APR-246 activates the TP53 pathway in MDS/AML cells with TP53 mutations, induces apoptotic transcription, and enhances the antitumor effects of azacitidine (Lehmann et al., 2012). The new study found that APR-246 decreased intracellular GSH content and induced lipid peroxidation to occur, while the anti-ferroptosis mechanism in AML cells enhanced the anti-leukemic activity of APR-246. In addition, this study further revealed that APR-246 action is not limited to TP53 mutated cells, which allows treatment to be applied to a wider range of patients with AML (Birsen et al., 2022). A phase II clinical trial of APR-246 combined with azacitidine in patients with TP53-mutated MDS/AML yielded good results (Mishra et al., 2022).

### 5.3 ATPR

ATPR is a novel all-trans retinoic acid derivative (Li et al., 2017) that shows superior anti-cancer efficacy compared to ATRA, and is expected to be a novel drug for the treatment of cancer (Du et al., 2018). Previous studies have shown that ATPR induces cell proliferation and differentiation by ROS accumulation and that ATPR also induces apoptotic autophagy in leukemia (Du et al., 2018). A recent study found that ATPR increases cellular autophagy by increasing ROS levels, which in turn regulate iron homeostasis and Nrf2, causing ferroptosis. Targeting iron homeostasis simultaneously induces ATPR-induced differentiation of AML cells and inhibits cell proliferation (Du et al., 2020).

### 5.4 Neratinib

Neratinib is a tyrosinase inhibitor, which has good anti-tumor effects in lung cancer and breast cancer. Applying neratinib to AML can inhibit the proliferation of HL-60 cells, induce G0/G1 cell cycle arrest, and undergo cellular autophagic ferroptosis. Researchers further explored the mechanism of action of neratinib on AML cells and found that the addition of autophagy inhibitors could attenuate the ferroptosis induced by neratinib on HL-60 cells, suggesting that neratinib inhibits the proliferation of AML through autophagy-dependent ferroptosis (Ma et al., 2022).

### 5.5 Sulfasalazine

Sulfasalazine is an anti-inflammatory drug that induces ferroptosis in cancer cells by inhibiting the function of SLA7A11 and preventing GSH synthesis (Zhu et al., 2022). Similarly, application of the systemic XC inhibitor sulfasalazine to AML cells decreases GSH levels and GPX4 activity (Zheng et al., 2022), inducing ferroptosis in the cells. Moreover, the synergistic action of sulfasalazine with anthracyclines and erythromycin can increase the anti-leukemic activity (Pardieu et al., 2022).

### 5.6 Iron complexes

Chlorido [4-carboxy-1,2-disalicylideneaminobenzene]iron (III) is an iron complex consisting of anti-leukemic activity of the same anti-leukemic effect of chlorido [N, N′-disalicylidene-1,2- phenylenediamine]iron (III) with the introduction of a 4-COOH group in the 1,2-phenylenediamine portion (Baecker et al., 2020), both of which are anti-AML cell metabolism and proliferation via ferroptosis (Sagasser et al., 2019), chlorido [4-carboxy-1,2-disalicylideneaminobenzene]iron (III) was more active than chlorido [N, N′-disalicylidene-1,2-phenylenediamine]iron (III) and also showed a good effect on perphenazine and erythromycin-resistant AML cells.

### 5.7 Artemisinin derivatives

Artemisinin is an antimalarial drug with more than ten derivatives including artesunate and dihydroartemisinin DHA, whose drug activity is mediated by ROS induction, has a remarkable anticancer effect, and performs excellently in AML cells (Kagan et al., 2021; Zhang et al., 2022b). DHA is an FAD-approved antimalarial drug with good anticancer efficacy against different human cancer cells. Previous studies have shown that DHA induces cancer cell death via ferroptosis in head and neck tumors and pancreatic cancer (Lin et al., 2016; Du et al., 2019). An experimental study on AML cells showed that DHA-induced autophagy in AML cells affected the AMPK/mTOR/p70S6k signaling pathway, degraded ferritin, increased the intracellular unstable iron pool, accumulated large amounts of ROS, induced lipid peroxidation, and ultimately led to ferroptosis (Du et al., 2019). A further study found that DHA induced early ferroptosis by promoting ferritin phagocytosis and iron overload and activated zinc metabolism to upregulate metallothionein and regulate GSH content, effectively promoting ferroptosis in AML cells (Grignano et al., 2023). Artesunate has a significant inhibitory effect on AML cells, and some experiments have shown that the anti-AML cellular activity of artesunate mediates cell differentiation mainly by modulating the ROS/Bim pathway to induce apoptosis and by relying on TFR cells to regulate intracellular iron homeostasis. Thus, artesunate derivatives have the potential to induce ferroptosis in AML cells (Liu et al., 2023c).

### 5.8 Typhaneoside

Typhaneosides extracted from *Cyperus rotundus* pollen have unique pharmacological activities. Zhu et al. found that TYP significantly inhibited the proliferation of AML cells and stalled the cell cycle in the G2/M phase. TYP-treated AML cells undergo autophagy, cause mitochondrial damage, and significantly increase intracellular ROS production (Zhu et al., 2019). It has been shown that autophagic signaling induces ferroptosis through ferritin degradation (Hou et al., 2016). Subsequently, Zhu et al. subsequently found that TYP enhanced lipid peroxidation, decreased GSH and GPX4 activity and induced ferroptosis in AML cells. At the same time, TYP did not affect normal cells, indicating that TYP is a low-toxicity and an efficient inducer of ferroptosis.

## 6 Conclusion

**FIGURE 4:**
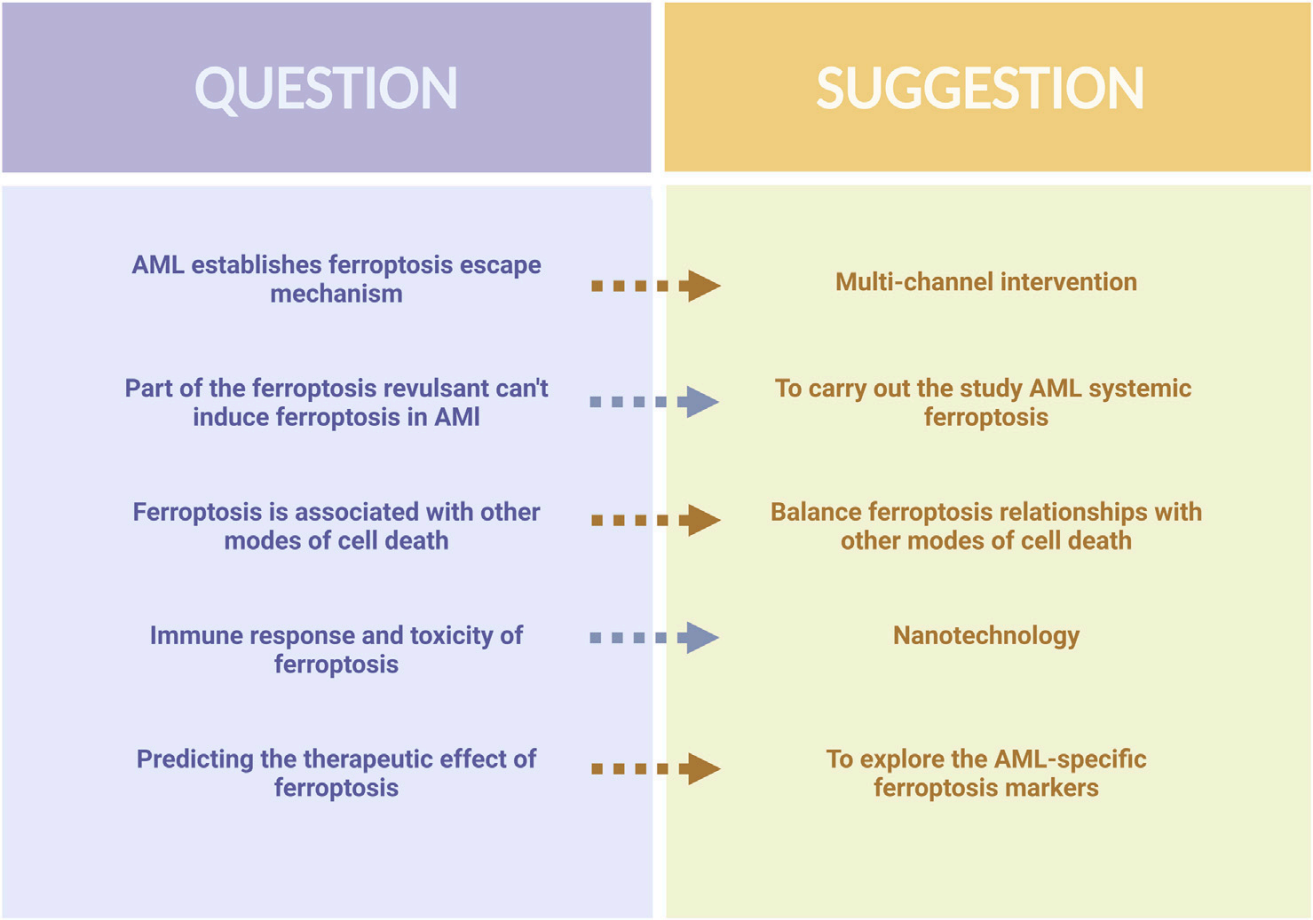
Difficulties and recommendations in applying ferroptosis to AML treatment.

In recent years, the study of ferroptosis in AML has made significant progress. This new form of cell death differentiates itself from conventional cell death and holds great potential for anti-tumor applications. Metabolic alterations in AML cells, as well as genetic changes like IDH and FLT3, create a favorable environment for ferroptosis to occur. Several molecular targets closely associated with AML, including SLC7A11, FSP1, GPX4, and DPP4, have been identified through the construction of AML models (Song et al., 2021). Furthermore, certain drugs such as APR-246, ATPR, and other natural compounds have been discovered to induce ferroptosis in AML cells. As ferroptosis is closely linked to the metabolism and oxidative stress of AML cells, exploring the relationships between ferroptosis and the pathogenesis, progression, and drug resistance of AML could shed light on new avenues for treatment. Nevertheless, the established ferroptosis defense system of AML cells adds uncertainty to the occurrence of ferroptosis and the application of ferroptosis in AML treatment faces some difficulties (Figure 4).

First, based on the available literature, we believe that ferroptosis in AML is a multiparty process. The induction of ferroptosis by a single pathway is likely to evoke a ferroptosis evasion mechanism in AML, leading to failure of the ferroptosis induction process. The paucity of available drugs targeting ferroptosis in AML cells also supports this. Therefore, the combined multi-pathway inhibition of ferroptosis evasion mechanisms should be sought to ensure that ferroptosis can occur successfully. Second, drugs such as Sorafenib and Brusatol have been validated as effective inducers of ferroptosis in some cancers (Tang et al., 2023; Ballout et al., 2022); however, they do not cause AML cell death by inducing ferroptosis, and it is necessary to dig deeper into the mechanism of the inability of ferroptosis to occur with these drugs, and to carry out a systematic ferroptosis study for AML. Third, there are interactions between ferroptosis and other cell death pathways, such as ferritin degradation associated with autophagy, increased iron and ROS levels (Hou et al., 2016), and the activation of excess ROS, which may cause cellular autophagy and chemotherapy resistance (Gu et al., 2020). Therefore, balancing these relationships is important. In addition to its association with cancer, ferroptosis is closely linked to pathological cell death in neurodegenerative and ischemic diseases, and the induction of ferroptosis may lead to additional toxicity and injury. At the same time, ferroptosis can also modulate inflammatory and immune responses (Zhong et al., 2022), which may pose challenges in AML treatment. Therefore, we believe that the development of nanotechnology allows iron NP inducers to precisely target cancer cells to avoid systemic adverse effects while inducing ferroptosis in AML cells (Liu K. et al., 2023). Further exploration of labeled AML-specific ferroptosis markers could accurately predict the effectiveness of ferroptosis therapy and improve the potential of ferroptosis in clinical applications.

By reviewing the alterations in metabolism and gene mutations that generate the susceptibility of AML cells to ferroptosis, this article provides insights into the therapeutic strategies for AML. It also highlights the coping strategies established by AML cells to avoid ferroptosis, as well as the potential therapeutic options against ferroptosis. Although the understanding of the pathogenesis and drug resistance mechanisms of AML from the perspective of ferroptosis remains to be improved, the application of ferroptosis to AML, including small molecule inhibitors, natural compounds, and nano-delivery of drugs may be a new direction for the treatment of AML in the future.

## Figures and Tables

**Table:** 

**2HG**	(R)-2-hydroxyglutarate
**AA**	arachidonoyl
**ACSL4**	Acyl-CoA synthetase long-chain family member 4
**ALDH3A2**	aldehyde dehydrogenase 3a2
**ALL**	acute lymphocytic leukemia
**AML**	Acute myeloid leukemia
**BDH2**	3-hydroxybutyrate dehydrogenase-2
**BHA**	tetrahydrobiopterin
**COQ10**	coenzyme Q10
**COX**	cyclooxygenase
**COX2**	Cyclooxygenase 2
**CYP**	cytochrome P450 monooxygenase
**DAMPs**	damage-associated molecular patterns
**DHA**	Docosahexaenoic acid
**DHODH**	dihydroorotate dehydrogenase
**DMT1**	divalent metal transporter1
**ECT**	electron transport chain
**EPA**	eicosapentaenoic acid
**ESCRT-III**	endosomal sorting complexes in retrograde transport-III
**FLT3**	Fms-like tyrosine kinase 3
**FPN**	ferritin-ferromodulin
**FSP1**	ferroptosis regulatory proteins
**FTH/FTL**	Ferritin heavy/light chain
**GCH1**	GTP cyclohydrolase 1
**GSH**	glutathione
**GSS**	glutathione synthetase
**HMGB1**	High mobility group box 1
**HO-1**	heme oxygenase-1
**HSCT**	hematopoietic stem cell transplantation
**IDH1/2**	isocitrate dehydrogenases 1/2
**JNK/c-Jun**	C-Jun N-terminal kinase
**KG**	ketoglutarate
**LCN2**	Lipid transport protein-2
**LIP**	labile iron pool
**LOXs**	lipoxygenases
**LPCAT3**	lysophosphatidylcholine acyltransferase 3
**LSCs**	leukemic stem cells
**MAPK**	mitogen-activated protein kinase
**MUFA**	monounsaturated fatty acyl
**NCOA4**	nuclear receptor coactivator 4
**NF-κB**	nuclear factor kappa B
**NGAL**	neutrophil gelatinase-associated lipid transport protein
**NOS**	non-coupled nitric oxide
**NOX**	nicotinamide adenine dinucleotide phosphate (NADPH) oxidase
**Nrf2**	nuclear factor erythroid 2-related factor 2
**PLA2**	phospholipase A2
**PLOOH**	phospholipid peroxides
**PLs**	phospholipids
**PUFAs**	polyunsaturated fatty acids
**ROS**	reactive oxygen species
**SAPKs**	stress protein kinases
**SCD1**	Stearoyl-CoA desaturase 1
**SLC3A2**	solute carrier family 3 member 2
**SLC7A11**	solute carrier family 7 member 11
**SODs**	Superoxide dismutases
**TAG**	triglycerides
**TF**	Transferrin
**TFR**	transferrin receptor
**USP15**	Ubiquitin-specific peptidase 15
**VHL**	Von Hippel Lindau
